# ‘Information on the fly’: Challenges in professional communication in high technological nursing. A focus group study from a radiotherapy department in Sweden

**DOI:** 10.1186/1472-6955-11-10

**Published:** 2012-07-23

**Authors:** Catarina Widmark, Carol Tishelman, Helena Gustafsson, Lena Sharp

**Affiliations:** 1 Dept. of Learning, Informatics, Management and Ethics, Karolinska Institutet, Stockholm, Sweden; 2 Dept. NVS, Division of Nursing, Karolinska Institutet, Stockholm, Sweden; 3 Foundation Stockholms Sjukhem, R& D unit, Stockholm, Sweden; 4 Dept. of Oncology, Karolinska University Hospital, 171 76, Stockholm, Sweden

## Abstract

**Background:**

Radiotherapy (RT) units are high-tech nursing environments. In Sweden, RT registered nurses (RNs) provide and manage RT in close collaboration with other professional groups, as well as providing nursing care for patients with cancer. Communication demands on these RNs are thus particularly complex. In this study, we aimed to better understand problems, strengths and change needs related to professional communication with and within the RT department, as a basis for developing a situation-specific intervention.

**Methods:**

Focus groups discussions (FGDs) were conducted with different professional (RNs, assistant nurses, physicians, engineers and physicists) and user stakeholders. Transcripts of the FGDs were inductively analyzed by a team of researchers, to generate clinically relevant and useful data.

**Results:**

These findings give insight into RT safety climate and are presented under three major headings: Conceptualization of professional domains; Organization and leadership issues; and Communication forms, strategies and processes. The impact of existing hierarchies, including how they are conceptualized and acted out in practice, was noted throughout these data. Despite other differences, participating professionals agreed about communication problems related to RT, i.e. a lack of systems and processes for information transfer, unclear role differentiation, a sense of mutual disrespect, and ad hoc communication taking place ‘on the fly’. While all professional groups recognized extensive communication problems, none acknowledged the potential negative effects on patient safety or care described in the FGD with patient representatives. While RNs often initially denied the existence of a hierarchy, they placed themselves on a hierarchy in their descriptions, describing their own role as passive, with a sense of powerlessness. Potential safety hazards described in the FGDs include not reporting medical errors and silently ignoring or actively opposing new guidelines and regulations.

**Conclusions:**

There is a risk that RNs who view themselves as disenfranchised within an organization will act with passive resistance to change, rather than as change promoters. As interventions to strengthen teams cannot be stronger than the weakest link, RNs may need support in the transition “from silence to voice” in order to take a position of full professional responsibility in a multi-professional health care team.

## Background

### Introduction and aim

As a first step in developing an intervention to improve team communication, a series of focus group discussions (FGDs) were conducted to take inventory of communication problems experienced by different stakeholder groups involved in radiotherapy (RT). In the study presented here, we aimed to investigate how these different stakeholders at one oncology department in Sweden discuss communication-related aspects of the domain of RT Registered Nurses (RNs) as well as the RT organizational structure and communication within and around the RT unit. We wanted to better understand problems, strengths and change needs related to communication, as a basis for developing a situation-specific intervention.

We discuss these context-specific findings in regard to their implications for the role of the RN in patient safety, multi-professional teams and processes of change in general.

### Contextual background

RT units are unusually extreme high-tech environments for nursing care, which impact not only on patients and family^1^ but also on the staff working at such units [1]. In Sweden, RNs with one year post-graduate RT education provide and manage RT, rather than this being the domain of medical doctors (MDs) or technical staff as is the case in most countries. As RT RNs work in close collaboration with physicists, MDs, assistant nurses and engineers, this also means that the communication demands on these RNs are particularly complex.

Communication demands, as well as inter-professional collaboration and teamwork is also argued to be more challenging in situations in which professional boundaries are protected, a phenomenon Weller refers to as *tribalism*[2]. Powell and Huw points out that this can jeopardize patient safety [3], whereas Braithwaite et al [4] note that professional rivalry and distrust is particular difficult to influence.

In addition, patient contacts common to most nursing roles, RT RNs must understand and convey messages about technical aspects of RT, physics, and wide varieties of nursing issues—including a multitude related to cancer as a potentially life-threatening disease—to patients and their families as well as to many diverse professional groups, often simultaneously. In this high-tech environment, patient safety issues are also crucial. There have been estimates that injuries are about one thousand times more common during RT than they are in relation to commercial air flights [5].

### Situation-specific background

Oncology care in the Stockholm region was previously provided at two university hospitals, by two geographically separate and distinct departments with very different organizational and professional sub-cultures and an often competitive relationship between sites. The politically-initiated merger of these hospitals into one in 2004 [6] was a notably difficult process for the oncology department as a whole. In contrast to many other areas in the oncology department, the leadership of the joint RT unit, both overarching and at the different geographical sites, stated that they found many potential benefits in the merger for patient care (e.g. in eliminating the long waiting times for RT) and were committed to finding ways to work together.

In efforts to facilitate collaboration across RT sites with the long-term goal of improving patient safety and care, a project group was formed to address RT nursing-related communication problems, which might be detrimental to patient safety. The project team was composed of RN clinicians and managers from within the RT department, and external academics and researchers. The data presented here derives from the first component of a nursing-focused action-research project to improve communication at the RT department, and provided a foundation for planning a situation-specific intervention.

## Methods

As noted above, the study presented here represents a first stage in a broader action-research project aiming to facilitate better communication and thereby minimize threats to patient safety. FGDs were conducted with a variety of staff and patient stakeholders, to increase our understanding of problems, strengths and change needs related to communication with and within the RT department from different perspectives, prior to detail-planning a situation-specific intervention. We deemed this approach appropriate, as FGDs encourage discussion among participants and thus provide information that might not be accessible in an individual interview situation [7]. Group processes can help people to explore and clarify their views and can encourage participation from those who feel that they have little to say [8]. Based on previous experience with FGDs with health care staff [9,10] we also thought discussing in a group context rather than in individual interviews might increase the potential for critical views about the workplace to be aired.

The members of the research team with clinical affiliations invited staff members to participate in FGDs. Staff were purposefully chosen to include those known to have strong opinions and/or represent perspectives from different teams and sites working at or in contact with the RT department. Four FGDs were homogeneous in the sense that they consisted of nursing staff only and were not mixed by site, to enable us to better understand nursing-specific as well as site-specific perspectives. The research team formulated a guide to be used to inspire discussion, which is presented in Table 1. Table 2 provides information on the constitution of the FGDs.

**Table 1:** Discussion guide for FGDs with professionals

♦	Describe the chain of command at your work place
♦	If you encounter problems during work, who do you contact?
♦	How do you know who to contact in case of problems—is there ever any doubt who to
♦	Describe the chain of command at your work place
♦	If you encounter problems during work, who do you contact?
♦	How do you know who to contact in case of problems—is there ever any doubt who to contact?
♦	What is usually most disturbing for you during a working day?
♦	When there are problems with communication, who do you feel is usually most affected?
	Prompt: You? the patient? other parties?
♦	Describe an incident that was troublesome to you related to communication?
	Prompt: What happened? who was involved? how would you have liked the incident to have been handled?
♦	Describe an incident with successful communication?
	Prompt: What happened? who was involved? what made the incident successful?
♦	Describe the leadership at your workplace
	Prompt: What do you think their role is? is concern for your work situation shown? if so, please describe
♦	Has the situation changed after the merger of the two departments? If so, please describe how
	Prompt: do you think communication problems you have described have improved? have they escalated?

**Table 2:** Focus group composition

**FGD #**	**SITE**	**PARTICIPANTS**	**NR. OF PARTICIPANTS (n = 34)**
1.	Y	RT RNs	7
2.	X	RT RNs/assistant nurses	8
3.	Y	RNs/assistant nurses in other sections of the oncology department	3
4.	X & Y	MDs, physicists & engineers	6
5.	--	Patient representatives from three cancer patient organizations	5
6.	X	RNs/assistant nurses in other sections of the oncology department	5

The FGDs were conducted during RT working hours at both hospital sites to ease participation and were audio-recorded after receiving written informed consent from participants. Light refreshments were provided during the FGDs, which lasted approximately two hours each, to encourage an informal atmosphere and compensate for any missed breaks. The FGDs were conducted in a conversational manner, with participants encouraged to raise relevant issues. These issues then contributed to adjustment of the guide for the following FGDs. Four FGDs were moderated by, a PhD prepared midwife experienced in FGD research but with no prior knowledge of the oncology or RT department, which allowed her to pose naïve questions. An additional moderator, with no formal relationship with the oncology or RT department, but with a background in cancer care research, contributed to the FGDs with non-nursing staff and with patient representatives.

Prior to beginning the discussions, all participants were asked to respect confidentiality within the group, informed that the FGD would be transcribed verbatim, and that all names and identifiers would be removed prior to distribution to the research team; they were nonetheless cautioned to consider that despite this, they might be recognizable to clinical team members and managers. This project satisfactorily underwent ethical review as part of a larger project entitled: *Innovation Systems for Better Health* (Dnr 2008/623-31) by the Regional Ethical Review Board, Stockholm.

### Data analysis

Prior to analysis, efforts were made to anonymise the FGDs by removing data which could identify the participants. Data analysis began with a naïve reading and content summary of each FGD by a team member not involved in data collection, based on principles from Lindseth et al [11]. These summaries were then read and discussed by the project team, and served to highlight salient issues for focus in further analysis.

Continued analysis was carried out by the team members in conjunction with an external doctoral student in the field of organizational culture and change, affiliated with the above-mentioned *Innovation Systems for Better Health* project. The five FGDs with staff underwent more detailed coding and categorization by this group, working intensively at three research retreats. Each participant in a FGD was given a colour code in the transcript, to highlight group interaction and facilitate analysis of discussions. All text related to the same topic was inductively sorted into the following initial categories: interaction between professional groups; organizational features; communication; descriptions of professional roles (e.g. RNs, assistant nursing staff, MDs, engineers, and physicists); hierarchies; and leadership. As the content of the FGD with patient representatives differed from those with staff, relevant portions of this FGD were analyzed hermeneutically rather than by detailed categorization. The text in the categories was then reviewed by the analysis group, with salient analytic points and themes distinguished.

Quotes, in italics, are presented which typify the findings. False starts, repetitions, etc. have been removed to ease reading, but all effort has been made to retain the meaning of the comments. Quotes were translated from Swedish to English by the authors, who are all bilingual. Omitted phrases are indicated by …, while brackets [ ] indicate authors’ comments.

## Results

We present the analysis of the FGDs under the following three major headings: Conceptualization of professional domains; Organization and leadership issues; and Communication forms, strategies and processes. The impact of existing hierarchies, including how they are conceptualized and acted out in practice, was noted throughout these data.

### Conceptualization of professional domains

#### RT RNs’ perspectives of their domain

One of the main challenges the RT RNs described in their professional role was the integration of humanistic patient care into the high-tech RT environment. The RT RNs said they had too little time for nursing care in the 10-minutes allotted to each patient, although they described themselves as “*specialists in the short encounter*” (FGD #1). They said this problem was somewhat compensated for by having special clinics for particular patient problems (e.g. for patients with head and neck cancers experiencing eating difficulties, weight loss, pain, etc). On the other hand, throughout the FGDs, RT RNs’ descriptions of an ideal day did not include reference to patient care, but involved: all technological equipment working without problems; patients arriving on time; full staffing without sick leave or other absences; and a well-prepared RT preparation process for each patient, completed in advance. Although this description is consistent with the complexity of the RT RN role noted above, it points to difficulties in maintaining a patient focus, highlighting the ‘conveyor belt’ organization of RT care.

This organizational complexity may be one factor involved in the sense of a general passivity and resignation expressed by the participating RT RNs. RNs were unanimous in their conviction that they lacked possibility to change their working conditions and had little or no impact on decision-making. When discussing how decisions were made, one RT RN said:

RN1: “*It feels like I stand…pretty far down on the hierarchical ladder. I’m the one who works, works, works, works all the time, all day long, but I make no decisions…I’m not involved and have no influence in any way…someone else has to influence things for me”* (FGD #2).

This nurse continues after a few minutes in dialogue with a colleague:

RN1: “*I think that there are pretty many decisions that I don’t have the possibility of influencing…sometimes it feels a bit powerless actually…And I think that I hear this from many of my colleagues”.*

RN2: “*It* [efforts to influence change] *will just fail anyway, and I think you…especially if there are a lot of patients, and people are stressed—then you don’t have the energy to deal with things, and especially if you know that you aren’t even going to be heard…”* (FGD #2).

This lack of power appears to be internalized by RNs, who describe a lack of agency in matter-of-fact terms as well. One frequently occurring example of this is that RT RNs often identified themselves in terms of the geography of their workplace, personifying the “*corridor”*, “*RT room*” or “*machine”* with which they work: ”*Some rooms are more generous than others”* or “*we have rooms that don’t understand why we book more patients”* (FGD #2).

It is also notable that in all the FGDs with RNs, there is an initial explicit denial of the existence of a hierarchy between professional groups working at the RT units, although the language used tends to reinforce hierarchical thinking, e.g. *“when doctors decide something amongst themselves, there can be poor information comingdownto us, but they have so very much to do*” (FGD #6). This is further reinforced by a RN, external to the RT unit but with a position demanding close contact with the unit, who explains the role of the nurse in general without questioning: *“We ease the jobs of other and are praised for that”* (FGD #3).

### Other professions’ perspectives of RT RNs’ domain

The RT RNs’ ambition to integrate good patient care with their provision of high-tech treatments was not always supported. In the FGD with other professionals, one MD indicates both through words and tone of voice a blatant criticism of what is considered RNs’ undue pampering of patients, saying that:

*“Nurses are very keen that patients can come in almost right away and then leave. And they get the patients used to* [this]. *And then they attend to the patients so carefully. You should be able to work in a different way, and then take a real coffee break and relax”* (FGD #4).

This physician also describes the RT RN role in terms of “*RT machine hours*”, seeing the RN function as equivalent with treatment production, and the inclusion of nursing care in the RT unit as time-consuming and clashing with effective treatment of large numbers of patients. This tension is also expressed in other ways, not only through the words used, but also through a critical tone of voice and body language:

*…”when they* [RT RNs] *take the patients into the treatment room and when they are in the room, they talk to them and such, and afterwards as well. So I think there is a whole lot of time given to nursing care”* (FGD #4).

This MD describes RT RNs’ nursing role as primarily psychosocial. An assistant nurse states that many physical care needs are also not within the realm of the RT RN, instead describing the assistant role as a “*consultant*” (FGD #2) for RNs, including both assessment and management of e.g. skin reactions for patients undergoing RT.

On the other hand, the same MD as above also expresses—albeit indirectly—respect for the RT RNs’ competency and role, suggesting that doctors should have a mandatory auscultation with RT RNs each year, even if only to appreciate the importance of the physician responding quickly when the RN pages the MD.

Most tension is described between the groups of RT RNs and physicists, particularly at one RT site, due to what both professional groups describe as a lack of clarity and clear lines of demarcation between their professional domains. As one physicist says: *”…*[there are] *different ways of handling things … I don’t know exactly what the division* [of responsibility] *should be and what the rules are and so…”* (FGD #4).

The same physicist explains this further, saying:

“*in my experience, this is absolutely not an area of conflict. What I can see sometimes is that … RNs … think the physicists aren’t really actively initiating things, and so maybe the RNs start to do things that actually should be up the physicists”.*

In general, other professionals’ descriptions of RT RNs are not personally negative; RT RNs are seen on the whole as receptive to teaching, engaged in their work, and supportive of others, although there is limited congruence in RT RNs’ own conceptualization of their role and that expressed by other professional groups at the RT unit.

### Organization and leadership issues

As mentioned above, role differentiation was least clear between RT RNs and physicists. The RNs at one site often used derogatory language when describing interactions between these professional groups; this interestingly occurred at the RT site where RNs expressed their own professional position as strongest. RNs considered a variety of organizational reasons for this, as shown in this dialogue:RN1: “*They* [physicists] *have a very difficult role here with us, and I think they haven’t found their own position yet, where they should be…that’s the problem, that they don’t know which piece they should be involved in…”*RN2: “*But at brachytherapy they’ve managed, they found their role here, assignments and tasks are much clearer, who does what”.*RN3: “*And there theydosomething. Here with us they just stand around and watch”* [laughter].

RN4: “*And it can’t be much fun to just stand and watch and then sit in your room…maybe that’s the problem”* (FGD #1).

The moderator interjects here, asking if physicists function as consultants. A fifth RN responds, clarifying that that RNs have traditionally been responsible for the RT set-up process, only contacting physicists if they deemed it necessary, but that new routines call for team involvement from the onset: “*Yeah, that’s the way we usually use them, if we have a problem, we call! They haven’t always been booked in advance, but now they say that they want advance notice…”*

It seems that this lack of clarity and overlap in responsibility is a complicating factor in the RT organization, and is also compounded by a lack of clarity in hierarchical relationships, as RN1 above also points out: *“And I would say the physicists are also fighting to try to have a higher position than that they actually have here with us…fighting their way up”.*

This lack of clarity in organization has a number of problematic effects. In addition to pointing out an ineffective use of resources, a sense of mutual disrespect is expressed by both groups, who make generalizations about categories of staff. Individual contacts with particular staff members are relied on for smoothing the way in problematic situations, rather than more stable organizational solutions.

Ineffective use of a wide variety of resources is a recurring theme in the FGDs. MDs complain that RNs are protective of their territory, rather than considering how RT as a whole might be reorganized to be most effective: *“We are lacking RT RNs, that’s a bottleneck today, how many ‘machine hours’ can we have? If we were to have more machine hours, more staff, we could get rid of the queues. But it is set in stone, that RNs protect their own”* (FGD #4).

On the other hand, RNs, MDs and engineers—although not physicists-- all express criticism of the general organization with different structures deciding rules, norms and policies for the different professions involved in the RT process in the FGDs, although RT needs to be tightly coordinated to run without hitches. In addition, unanticipated service needs for machinery cause problems, with engineers critical of RNs’ lack of understanding of the engineer’s role in addressing such problems.

RNs are recognized to be the ones left to deal with the patient, when RT preparatory work is not completed in time, machines malfunction, and other professionals are delayed or remain absent. RNs describe being caught between their loyalty to the MDs they work with and patients’ needs for access to RT; the RNs describe providing care and service for both these parties, but not for engineers or physicists.

We had expected leadership issues to be a more prominent part of the FGDs; such discussions instead focused on informal leadership and formal management. As one RN criticized:

"“*We have invisible leaders. The managers are sitting far from RT and walking around in their private clothes and only come around for guest appearances…the head nurses that is, who barely have an idea of what we do in the treatment rooms”* (FGD #1)."

Despite these criticisms there was a lack of consensus about the need for visible leadership, with some arguing *“the tougher the situation, the more important with good leadership”* (FGD #3) and others stating: *“it’s good for us not to have a boss there on a daily basis”* (FGD #2). On the other hand, there was agreement in a lack of faith in the complicated organizational hierarchy, with multiple levels of managers with little clinical knowledge and experience of the workings and needs of the RT unit. But this situation was also seen to leave a vacuum, allowing the proliferation of informal leaders among MDs and physicists, with backing from previous department heads. Informal leadership is thus accepted and institutionalized with parallel structures developing, instead of changes proposed in accordance with the hospital-wide first-line management policy newly in place. This situation may be in part in response to the merger between hospitals, as a means of maintaining existing power structures informally. An example of this is one MD declaring himself to be *“the extended arm of the department head—his eyes and ears”* (FGD #4).

The development of informal structures is only one of the remaining effects of the hospital merger. This is evidenced, e.g. by differences in nursing culture, with one site being called “*hell”* and the other “*heaven”* by a RN with work experience from both. A remaining hierarchy was also described, with one site said to be favored: *“X site decides…all the bosses come from X site”* and slurs about medical breadth and competence at the other site. One the other hand, some participants in the FGDs do acknowledge that after several years, a shared system with benefits for RT staff and patients has been developed. Other staff point out tensions that also exist between different units on the same geographic site, and even between different teams on the same unit.

### Communication forms, strategies and processes

Communication is generally described as based on ad hoc behaviors and solutions in impromptu encounters, rather than occurring through systematic and planned forms and processes. Information within and between professional groups is described as occurring ‘on the fly’. This appears not to be a matter of choice, but a result of the lack of structures for information transfer. As one RN says:

*“…there is this ‘flying information’ that I am totally allergic to. That you get new information at the same time as you are doing other things. You aren’t receptive…what I miss are short sittings, don’t have to have many, maybe just for 10 minutes, those of us working together today, so that you can raise issues that—yeah, important things, and that it is done repeatedly”* (FGD #1).

A second RN agrees, adding:

“That’s just what we experience working at our machine also, when you are running a treatment and someone can come in and interrupt. In my experience, you can’t concentrate on more than one thing at a time”

Poor systems, or in some cases, lack of systems for communicating information are said to take several forms, including second-hand information, spread of unsuitable information, information that misses parts of the RT organization, etc. RNs also describe a feeling that the organization “*has a low ceiling”* and censures discussion. A particular problem discussed by several professional groups was a lack of clarity around decision-making, with information about new decisions and policies often spread in what several people metaphorically called “*Chinese whispers”* or “*playing telephone”* (FGD #2) referring to children’s party games. This was said to lead not only to a lack of transparency, but also the lack of acknowledgement of mandates for implementing change. Notably, there was even debate about whether common meeting forums did in fact exist, which could then serve to improve communication. At one site, FGD participants complained that even informal meeting places were lacking, which they felt might have compensated for the lack of formal forums.

Another notable communication difficulty was that the professions involved in RT used language differently. The most extreme example of this was the lack of consistent meaning in describing right and left in the RT treatment field. Right and left is described from the perspective of the patient’s own body by MDs but these designations change with different patient positions. The other professional groups instead use the terms right and left to refer to placement in the treatment room. The implementation of a new digital verification system demands that MDs change their language use to adapt to that used by other professional groups, but this was said to be met with resistance.

This type of resistance was also common among RT RNs, with numerous examples matter-of-factly provided by the RNs. In the FGDs a number of different situations and strategies for silently opposing new regulations were noted, e.g. not reporting medical errors—a behavior shared by the other professionals—, or not contacting physicists or MDs in accordance with regulations in routine situations deemed unproblematic. This ‘silent’ communication of dissent was repeatedly described, whereas few situations of open, verbal resistance or discussion were provided in the FGDs. Situations in which RT RNs describe themselves as directly confrontational were most evident when RNs felt patients were treated disrespectfully by other professionals, e.g. when MDs or physicists enter a treatment room and approach an undressed patient without presenting themselves.

Yet another feature of communication which was raised by patient representatives in a FGD, as well as by various professionals, was the ‘silo’ organization of cancer care in general, including RT. This meant that RT teams functioned as “*cocoons”* often not involved in other aspects of the RT department, but also that information to patients was fragmented and relevant only to specific units. One patient representative explained:

*“I have a lot of experience of the way information around patients is handled, and it rarely follows the patient’s path through the health care system. It is instead more focused on units, the different specialties and so on, but for the patient, cancer care is a new world they have to orient themselves to, and therefore it is important that information structures also follow the patient’s pathway throughout the trajectory”.* (FGD #5)

In general, professionals repeatedly stated that patients remained oblivious to the organizational and communication problems discussed in the FGDs. However it was clear from the FGD with patient representatives, that patients were indirectly affected by many of the issues discussed by professionals.

## Discussion

It is notable that despite other differences, the professionals participating in these FGDs presented a common picture of communication problems related to RT, i.e. a lack of systems and processes for information transfer, unclear role differentiation, a sense of mutual disrespect, and ad hoc communication taking place ‘on the fly’. While all professional groups recognize extensive communication problems, none acknowledge the potential negative effects on patient safety or care which are described in the FGD with patient representatives. Potential hazards described in the FGDs include not reporting medical errors and silently ignoring or actively opposing new guidelines and regulations. While these data are generated from a limited number of focus groups in a specific RT context, we argue that many of the findings and their implications may well be relevant in other—especially high-tech and multi-professional—acute care nursing settings.

Through analysis of these FGDs, we indirectly gain insight into both the safety climate and the safety culture on the RT unit, although this was not the original aim of the project. Sexton et al^1^ differentiate between the terms *safety culture* and *safety climate*, although pointing out that the terms are frequently used interchangeably in health care. They define safety climate as “the consensus of shared perceptions regarding patient safety, and norms and behaviors by frontline workers in a given clinical area” (p 935). Safety culture on the other hand, according to Sexton et al [12], demands deeper investigation, through “careful and time consuming observation of norms, beliefs, values, artifacts, symbols, and rituals” (p 934). Safety climate may thus change relatively quickly over time to reflect new routines and interventions, for example after a medical error [13]. Safety culture, however, is more deeply embedded and less susceptible to fluctuation or change, as it represents unwritten, rather than formalized rules and regulations. In this study, the FGD participants’ descriptions suggest that both safety climate and safety culture are negatively affected by the above-noted communication deficits.

It should be recognized that this FGD study does not use approaches which according to Sexton et al [12] would be optimal to in-depth investigate the symbols, rituals and values intrinsic to safety culture. FGDs on the other hand, do generate data about shared perceptions which illuminate safety climate and shed some light on norms and beliefs common to a workplace. There are some factors to consider when interpreting these data however. One is the extent to which existing clinical and workplace hierarchies can be mirrored in some FGDs, with less input from the person further down on a perceived hierarchy. While this was not noted between the RNs and assistant nurses in the FGDs, it may have had more impact in the FGD with other professionals, with the informal leadership role of one participant apparent.

Using evolutionary psychology, Braithwaite et al [14] present conceptual underpinnings of communication breakdown in health care situations, including their potential negative impact on patient care. They summarize the problem saying “when organizational failure looms, trust and communication are compromised” (p 354), arguing that effective communication and trust between professional groups are essential for a well-functioning multidisciplinary team. Braithwaite et al [14] mean that managers in health care settings rarely succeed in uncovering existing underlying issues and when they do, that poor communication and lack of trust results in the managers often being ignored by staff, which tends to protect their own interests before that of patients. From another perspective, in their analysis of major health care ‘failures’ from six countries, Walshe and Shortell [15] summarize common themes as related to 1) longstanding problems which are 2) well-known but not addressed and 3) which can cause immense harm. These often occur in 4) dysfunctional organizations, lacking basic management systems or with systems that are readily bypassed, with a lack of coherent clinical leadership, and with 5) some types of failures occurring repeatedly, thus indicating that “lessons are not being learned” (p 106).

In these FGDs, we both see evidence of the lack of trust pointed to by Braithwaite et al [14] and the five points highlighted by Walshe and Shortell [15]. RT RNs describe a basic mistrust of physicists, and a situation in which they feel that their professional competence is not trusted by other professionals. Accusations of professional territoriality are raised by different actors, often in relation to RNs, although this specificity may be an artifact of the study focus and design. Many, but not all managers are described as ineffectual and removed from a position allowing real insight into the more subtle problems and needs of the RT units and teams. There is shared recognition of problems, but they are longstanding and have not been adequately addressed, with formal efforts at change often bypassed by staff. The implications for these problems on patient safety, appears to be a lesson that has not been learned, to use Walshe and Shortell’s [15] expression.

Neither Braithwaite et al [14] nor Walshe and Shortell [15] directly broach the topic of hierarchies in health care systems, although this has permeated our FGD data both directly and indirectly. As previously noted, RNs tended to initially deny the presence of a hierarchy between professional groups, although they used language which indirectly indicated that these RNs placed themselves below physicians and above physicists (particularly on one site) in conceptualizations of their workplace, hierarchical roles, and responsibilities. This hierarchical relationship between RNs (primarily women) to physicians (mixed sexes) seems to be generally acknowledged (see e.g. classic work of Stein [16], further discussed in Stein et al [17]; Reeves et al [18]) and tacitly accepted by these RNs—particularly noteworthy in these Swedish data, from a country otherwise renowned for being relatively egalitarian. It can also be noted that little friction was described between the engineers and the RNs by either group, although engineers are generally seen to have higher status and have markedly higher incomes despite the same length and academic status in their educational programs, and the relatively high status (informal and in terms of salary) enjoyed by the RT RNs in comparison with other groups of their peers (e.g. compared to other RN specialists in high-tech environments).

Perhaps the most disconcerting feature of the hierarchical RT environment was the passive role described and assumed by the RT RNs participating in these FGDs in many situations. The RNs often express a lack of “voice” and a sense of powerlessness, but at the same time rarely describe an individual sense of professional responsibility. This pattern was described by Widmark et al [19] over a decade ago, in regard to another relatively autonomous group of RNs that is midwives in Sweden.

It is important to consider how to constructively impact this situation with the problems and communication deficits noted in these FGDs. Recently, Buljac-Samardzic et al [20] identified and reviewed three categories of interventions to improve team effectiveness; training, tools and organizational interventions, although the level of evidence was noted to be generally low. Different types of team training were best documented, although the heterogeneity in studies precludes strong conclusions and the researchers end with a call for better fit between diagnosed problems and the interventions to address them, with more attention paid to context. Butterworth et al^2^ discuss capacity-building and capability in patient safety from a nursing perspective, using an example from England for inspiration. They discuss how RNs are ‘culture carriers’ and how their potential can be maximized through changes in nursing education, increased focus on RNs’ ways of working, and increased interest from research funders. Unfortunately, Butterworth et al [21] limit their discussion to albeit important, but very specific nursing issues, with little acknowledgement of the role RNs play in organizational settings with multi-professional interaction and the demands this places on communication.

## Conclusions

One question which remains is how to achieve not only changes in safety climate, but also in safety culture. Based on these data, we are concerned that constructive and sustainable change may not be possible through implementation of specific safety and/or communication tools alone, although it has been said that their repeated use will often also influence safety culture [13]. We argue that as long as RNs view themselves as disenfranchised within an organization, there is a risk that they will act with passive resistance to change, rather than as change promoters. Interventions designed to strengthen teams cannot be stronger than the weakest link in the team. Based on these data, we conclude that RNs may need support in the transition “from silence to voice” [22] in order to take a position of full professional responsibility in a multi-professional health care team.

## Endnotes

^1^ We use the term ’family’ in its broadest sense, to mean all significant others.

## Competing interests

The authors have no competing interests.

## Authors' contributions

CW, CT and LS made substantial contributions to the design and planning of the study. CW and CT led the FGDs. All four authors have contributed equally in analysis and interpretation of data. All four authors have been involved in drafting and revising the manuscript and have given final approval of the submitted version.

## Pre-publication history

The pre-publication history for this paper can be accessed here:

http://www.biomedcentral.com/1472-6955/11/10/prepub
